# Advances in Bio-Tactile Sensors for Minimally Invasive Surgery Using the Fibre Bragg Grating Force Sensor Technique:A Survey

**DOI:** 10.3390/s140406633

**Published:** 2014-04-09

**Authors:** Abdulfatah A.G. Abushagur, Norhana Arsad, Mamun Ibne Reaz, A. Ashrif, A. Bakar

**Affiliations:** Department of Electrical, Electronic and Systems Engineering, Faculty of Engineering and Built Environment, Universiti Kebangsaan Malaysia, 43600 UKM Bangi, Selangor 43600, Malaysia; E-Mails: norhana@eng.ukm.my (N.A.); mamun.reaz@gmail.com (M.I.R.); ashrif@eng.ukm.my (A.A.A.B.)

**Keywords:** fibre Bragg grating, optical tactile sensors, minimally invasive surgery

## Abstract

The large interest in utilising fibre Bragg grating (FBG) strain sensors for minimally invasive surgery (MIS) applications to replace conventional electrical tactile sensors has grown in the past few years. FBG strain sensors offer the advantages of optical fibre sensors, such as high sensitivity, immunity to electromagnetic noise, electrical passivity and chemical inertness, but are not limited by phase discontinuity or intensity fluctuations. FBG sensors feature a wavelength-encoding sensing signal that enables distributed sensing that utilises fewer connections. In addition, their flexibility and lightness allow easy insertion into needles and catheters, thus enabling localised measurements inside tissues and blood. Two types of FBG tactile sensors have been emphasised in the literature: single-point and array FBG tactile sensors. This paper describes the current design, development and research of the optical fibre tactile techniques that are based on FBGs to enhance the performance of MIS procedures in general. Providing MIS or microsurgery surgeons with accurate and precise measurements and control of the contact forces during tissues manipulation will benefit both surgeons and patients.

## 1. Introduction

Rapid developments have recently occurred in minimally invasive surgery, which has become a practical reality, especially after the advent of rod optics, optical fibres and the first solid-state cameras. The distinct advantages offered by MIS over conventional operations include reductions in the following: intraoperative blood loss, tissue trauma, risk of post-operative infection, pain experienced by the patient and recovery time [1]. However, there are two major drawbacks to such surgeries: the constrained spaces (only key-hole incisions are used), which lead to a reduction in the degree-of-freedom (DOF) during manipulation, and the absence of haptic feedback (including tactile forces) during the tool-tissue interactions [2,3]. Surgeons in MIS, including microsurgeries, must accurately and carefully manipulate delicate tissues using customised surgical tools (ranging from simple freehand to sophisticated tools) in constrained spaces. As a result, the surgeons may perform inappropriate tool movements and may suffer from premature fatigue during MIS [4,5]. Advances in robotic systems have made their use possible in the operating room, and minimally invasive robotic surgery (MIRS) systems are now common [6–8]. Consequently, robots in master-slave configurations, such as the ZEUS™ Surgical System [9] and the da Vinci™ Surgical System (DVSS) [10], have been introduced to solve motion-constraint problems in MIS. These systems have increased the attainable DOF of tool-tissue manipulation. This helps surgeons perform a variety of MIS operations more effectively for different types of abdominal interventions [11–15]. Nonetheless, the performance of the surgeons during MIRS or MIS manipulation is still severely limited by their having little to no tactile information compared with the rich tactile feedback of the human hand [16]. Artificial tactile sensors for mimicking human fingertips and tackling the mentioned drawback have been recently attracted the attention of many researchers, especially in the fields of robotics, telesurgery and medical rehabilitation [17–21]. Providing tactile feedback during tool-tissues interactions allows the surgeon to control the applied forces, thus preventing any tissue trauma or unintentional damage to healthy tissue [6]. In addition, distributed tactile information helps the surgeon to characterise, distinguish and investigate the contacted tissues; thus, better performance will be achieved.

In the past few years, several tactile sensors have been developed to provide tactile force information in MIS/MIRS and micro-surgeries. These sensors include the existing electrical strain gauges [22–27] and micro-electro-mechanical systems (MEMS)-based technology. MEMS technologies were introduced to replace electrical strain gauges as one step towards miniaturised force sensors. Examples of MEMS techniques include silicon-based sensors that use piezoresistive or capacitive sensing and polymer-based sensors that use piezoelectric polymer films (polyvinylidene fluoride); these films are well known, and PVDF films have been already demonstrated [28–32]. Although these sensors offer good spatial resolution, they still pose some problems, such as the wiring complexity, the rigid substrate and the fragile sensing elements [33]. In addition, most have an electrical base, which prevents their application in an MRI environment [34]. All these drawbacks can be overcome by using optical fibre-based sensors [35,36].

The inherent advantageous properties of optical fibres, such as the small size, immunity to the electromagnetic interference (EMI), biocompatibility, non-toxicity and chemical inertness, make the optical fibre an ideal alternative tactile sensor [37]. Various tactile force-sensing schemes based on fibre optic techniques have been investigated over the last several decades [38–40]. Optical fibre techniques are divided according to their sensing principle into three categories: intensity-modulated optical fibre sensors [41], interferometer-based optical fibre sensors [42], and FBG sensors [43].

Several fibre optic tactile force sensors that are based on the light intensity modulation technique has been developed for many MIS/MIRS applications. For example, a device containing three optical fibres that were arranged axially at 120° intervals was developed for MIRS [44]. The optical fibres were designed to measure the relative displacement between two parts of the device using the reflected light intensity signal. In another study [45], three optical fibres in a circle at 120° intervals were integrated into a catheter for cardiac catheterisation, thus providing an RF ablation catheter with force feedback. In a different study [6], a tactile sensor measured the light intensity modulation due to a micro-bending fibre that deflected the beam when normal forces were applied to the fibre. Another optical fibre tactile sensor in a 3 × 3 grid form was also developed [46]. The sensor was able to measure the change in the reflected light intensity due to a deflection caused by normal forces. Furthermore, the sensor device was able to be integrated into either an end effector for a robotic manipulator or an MIS application. Despite the low cost of the intensity-modulation technique, power fluctuations might affect the precision of the results due to the intensity.

In addition, techniques based on the Fabry-Perot interferometer (FPI) have shown great promise in this area. For example, a miniaturised fibre optic tactile force sensor utilising FPI has been proposed [47]. The sensor was designed to provide tactile feedback and measure the forces of interactions during needle-based percutaneous procedures in an MRI. In one report [48], a high-resolution fiber optic force sensor utilising FPI to measure the needle insertion force and provide a haptic display for use in brachytherapy applications was demonstrated. Another tactile sensor based on FPI has been reported in vitreoretinal microsurgery applications; this sensor simultaneously measured the axial and lateral forces [49]. However, the device was unable to measure the forces during a rapidly changing force input and required a complex detection system. The small size of the sensor also limited the working range of measurable forces for the technique.

MIS/MIRS or microsurgeries require tactile sensors to provide accurate information about features such as the force amplitude, direction and distribution profile under static and dynamic loading [50]. In addition, the required force sensing range can vary due to the range of mechanical properties of different tissue types. As a consequence, a tactile force sensor must cover the full-scale force range of the predefined tissues with which it will interact.

In the past few years, researchers have focused a great deal of attention on using the FBG technique for tactile force sensors. This rapidly emerging technique has applications in MIS/MIRS, catheter-based techniques and microsurgery. FBG has potential due to its distinct advantages over other fibre sensors, such as the use of the absolute wavelength as a sensing signal which implies that problems of light intensity fluctuations and limitations of phase discontinuities are avoided. In addition, the ease of multiplexing allows multiple independent sensors to be accommodated on a single fibre [51]. The use of FBG in medical applications is not new and has been proposed at the end of the past century and early in this century as a useful sensing device for temperature [52], pressure and strain detection [53,54], but its practicality for full-scale implementation has not yet been fully established [55].

Briefly, FBG is a distributed Bragg reflector constructed in a short segment of a fibre core by laterally exposing the fibre to a focused laser with periodic intensity. Due to this periodic variation in the refractive index, a certain narrow wavelength range will be reflected back, while the remaining wavelengths will be passed if broadband laser light is propagated through the fibre. The functional basis of the sensor is discussed briefly in the next section. FBG sensor technology show promise as this technique can meet all the previously mentioned requirements for MIS tactile applications. This technique inherently has a wide strain range that can exceed 8000 με with adequate resolution and sensitivity for both static and dynamic loads [56]. In one report [57], static and dynamic strain-induced wavelength shifts of the FBG sensor were measured. The experimental results demonstrated that a static strain resolution of better than 1 με and a dynamic strain sensitivity of $\approx 40\text{n}\varepsilon /\sqrt{\text{Hz}}$ at 50 KHz can be achieved with a proper interrogator system. Thus, the FBG sensor can cover force ranges from the microscale to macroscale under both static and dynamic loading. However, the major drawback of the FBG technology is the cross-sensitivity to the wavelength shifts caused by temperature and strain [58]; this problem is commonly solved by adding one mechanically isolated (strain-free) FBG for temperature compensation [59]. Table 1 list down the development of FBG tactile sensors in MIS applications.

This review covers the up-to-date on-going research of biotactile sensors that utilise an FBG-based sensor technique. The paper is organised as follows: the FBG working and sensing principles are briefly described in Section 2. In Section 3, two techniques using FBG tactile sensors and their different applications are discussed. Future perspective, if any, on the reviewed sensor designs has been briefly discussed in Section 4, followed by a conclusion.

## 2. FBG Working and Sensing Principle

In general, a Bragg grating in a fibre core results in back reflection of the guided light with a central wavelength that is given by [60]:

(1) $$\lambda_{B}=2n_{\text{eff}}\Lambda$$

where Λ is the pitch or periodicity of the grating, *n_eff_* is the effective refractive index of the core, and λ*_B_* is the Bragg wavelength. It is obvious from Equation (1) that the Bragg wavelength will shift if the effective refractive index or the grating periodicity changes due to any external perturbation. The wavelength shift due to a perturbation is a linear combination of the rate change of both parameters with respect to strain and temperature and can be calculated as follows [59]:

(2) $$\Delta \lambda_{B}=2\left[\Lambda \frac{\partial n_{\text{eff}}}{\partial l}+n_{\text{eff}}\frac{\partial \Lambda }{\partial l}\right]\Delta l+2\left[\Lambda \frac{\partial n_{\text{eff}}}{\partial T}+n_{\text{eff}}\frac{\partial \Lambda }{\partial T}\right]\Delta T$$

Where Δ*l* is the change in the grating length due to strain, and Δ*T* is any change in the ambient temperature. This wavelength shift provides the basis for strain and temperature sensing with typical values for the sensitivity to an axial strain and to temperature of approximately 1.2 pm/με and 13 pm/°C, respectively at a 1550 nm centre wavelength.

## 3. Tactile Sensor Types

Tactile sensing devices can be divided into two categories: tactile single-point sensors and tactile array sensors. The importance of the former in MIS/MIRS is to detect and measure only the amplitude or amplitude and direction of the contact forces during tool-tissue interactions. In contrast, tactile array sensors are utilised to measure spatially distributed forces along the sensor length or over a predefined sensor area, in which a concentrated force can be localised. In addition, some of the tactile array sensors are designed in a large grid pattern to detect the shape of the contacted object.

### 3.1. Single-Point FBG Tactile Sensors

**Figure 1.**
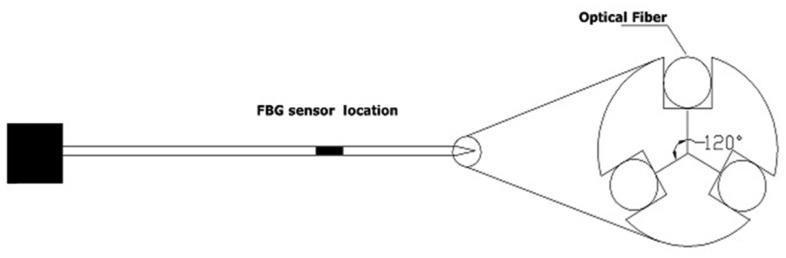
Schematic diagram of a single-point FBG tactile device with 2 DOFs.

A single-point FBG tactile sensor, as its name implies, consists of a single FBG sensor element located along a single optical fibre. The fibre then can be incorporated into a tool to measure the amplitude of the applied/contact forces. The single-point FBG tactile device can have more than one fibre arrangement to measure the force components of different axes. This section surveys and explains in detail the use of the FBG single-point technique to measure the peak force exerted during manipulation. The survey will describe two tactile devices with different configurations that are being developed in MIS/microsurgery applications. The first one is termed one-degree-of-freedom (1-DOF), in which with a single FBG element is integrated into a single fibre. The device can only function along one axis. In contrast, the second configuration is named 2-DOF, in which three optical fibres that each have a single-point FBG sensor element are arranged inside a device. Such design allows the device to measure two force components. Figure 1 depicts the schematic of the 2-DOF device.

#### 3.1.1. Vitreoretinal Microsurgery Applications

Many clinical procedures in MIS involve intervention into and manipulation of extremely small and delicate tissue structures. The forces exerted are either below the threshold of the surgeon's perception (e.g., microsurgery) or are exerted at the distal end of a handheld/robotic tool such that the surgeon will not feel the force; the applications of excess force will eventually damage the tissue. To avoid this damage, an artificial tactile sensor must be attached to or integrated into the tooltip of the surgical instrument, and this sensor must enable the host to acquire information about contacts with external objects. Retinal microsurgery is one example that requires micron-level manoeuvres. The manipulation of vitreoretinal structures inside the eye poses enormous challenges due to the tissue delicacy, the surgical inaccessibility, the suboptimal visualisation, and the potential for irreversible tissue damage from excess force or unintentional movement.

A research group at Johns Hopkins University has performed several consecutive research studies to design and develop a surgical instrument with a force sensor to address the challenges experienced during vitreoretinal microsurgical. Sun *et al.* [61] reported the initial development and preliminary evaluation data for a new family of force-sensing microsurgical instruments in early 2009. Their goal was to develop a force sensor that enables surgeons to measure the distal forces interior to the sclera. In the previously developed force sensor schemes, the electrical-based sensors were located outside the eye due to their size, in which discriminating between forces applied at the tooltip and forces from contact with the sclera was a challenge. Hence, due to the potential small size of the FBG and the other mentioned features, the researchers decided to utilise a single FBG in the tool shaft manipulator to design a 1-DOF tactile sensor that could measure the forces exerted at the tooltip while manipulating the inner surface of the eye. The tool was made of a titanium wire that was 50 mm in length and 0.5 mm in diameter to mimic a 25-gauge ophthalmic instrument. The length of the used FBG sensing element was 10 mm, and it was located 5 mm from the tool end for higher sensitivity. A laser wavelength interrogator was used to excite the FBG sensor element and analyse the returned spectrum. When the tool interacted with tissues, the exerted forces deflect the tool, causing the surface with the embedded FBG to experience either tension or compression, resulting in a wavelength shift.

The device was calibrated, and a linear relation between the force and wavelength shift was achieved with an adequate force resolution of 0.25 mN. During the calibration process, loads were applied to different positions of the device, starting with the point nearest to the tooltip. For loads that were applied 1 mm from the tooltip, the sensitivity of the sensor reached a peak of 14 pm/mN. Hence, forces of 70 μN could have been sensed if a FBG interrogator with 1 pm resolution had been used. This sensitivity continued to decrease as the loading points move further towards the other end of the tool. Table 2 depicts the relation between the sensitivity and the loading position of the tool.

To simulate realistic retinal vein cannulation and membrane peeling experiments, the researchers used a chorioallantoic membrane (CAM) from a 12-day-old chicken embryo for their analysis. This membrane was previously suggested [62] for use as an eye tissue model for surgical retinal research.

For comparison during the two manipulation experiments, the prototype force sensor was controlled in a freehand manner and with Eye Robot-assistance. The robot-assisted method showed a more stable signal and was less noisy than the freehand manipulation. This difference was attributed to the hand tremor effect. In addition, the force during the robot-assisted manipulation was slightly smaller than that during the freehand manipulation, indicating that the robot-assisted manipulation was more controllable. These experiments confirm that placing an FBG force sensor into the instrument shaft can measure extremely delicate forces while avoiding the confounding factor of tool-sclera interaction forces. However, the device only had a 1-DOF force sensor, which requires the surgeon to keep the tool in a certain direction during manipulations; this requirement is not practical for clinical use. In addition, temperature compensation was not considered.

Iordachita *et al.*, which was almost the same group and from the same university, published another study [63] that optimised the previous 1-DOF force sensor by inserting three fibres (instead of one) along the tool shaft with a single-point FBG sensor in each fibre near the tooltip. They placed the fibres evenly around the device with a separation of 120° (Figure 1). The goal of this 2-DOF device was to measure the forces in transverse planes (perpendicular to the z axis of the tool shaft). The design of the device required the development of an algorithm to eliminate the axial forces and the variations caused by the environmental temperature to measure the lateral *F_x_* & *F_y_* force components accurately.

The Bragg wavelength shift from (2) as a function of strain and temperature change can be expressed as:

(3) $$\Delta \lambda_{B}=K_{ɛi}{ɛ}_{i}+K_{Ti}\Delta T_{i}$$

Where *K_εi_*, *K_Ti_* are strain and temperature sensitivities; respectively. The subscript “*i*” indicates the sensor number. The parameters *ε_i_* and Δ*T_i_* are the strain applied to the sensor and temperature change around the sensor, respectively. The work assumed that the three FBG sensors experienced the same Δ*T* and that the temperature gradient along the surface of such a small volume could be neglected. The FBGs all utilised the same type of fibre; therefore, the temperature sensitivity *K_T_* was the same for all FBGs. Due to the linear relationship, the temperature effect can be cancelled from the three equations. Then, subtraction of the mean value of all three wavelength shifts from each wavelength shift led to the following sensor reading Δ*S*:

(4) $$\Delta S_{i}=\Delta \lambda_{Bi}-\frac{1}{3}\sum_{i=1}^{3}\Delta \lambda_{Bi}$$

Thus, the noises and axial forces could be eliminated. The values of Δ*S_i_*, the strain induced by the force components *F_x_* and *F_y_*, were expected to be linearly related, and the calibration matrix then was developed. During the calibration process, different forces were applied, and the induced sensor reading Δ*S_i_* was measured to solve for the calibration coefficients *K_iX_* & *K_iY_*.

(5) $$\left[\begin{array}{c} \Delta S_{1}\\ \Delta S_{2}\\ \Delta S_{3} \end{array}\right]=\left[\begin{array}{ll} K_{1X} & K_{1Y}\\ K_{2X} & K_{2Y}\\ K_{3X} & K_{3Y} \end{array}\right]\;\left[\begin{array}{c} F_{X}\\ F_{Y} \end{array}\right]$$

Therefore, the force components in an experiment could be calculated using the pseudo-inverse of the calibration coefficients *K*^+^ and the sensor reading Δ*S_i_*. The calibration was also performed by producing a heat source to observe the effect of the temperature on the unloading and loading of the device; this measurement also verified that the temperature affected all FBGs in exactly the same manner.

Two epiretinal membrane peeling experiments using the designed 2-DOF force sensors were planned. To mimic the hooks or microforceps that are often utilised in vitreoretinal surgery; a small hook was customised for attachment to the prototype device. This hook required the researchers to perform another calibration to determine new calibration coefficients. To simulate epiretinal membrane peeling, the researchers peeled and measured the forces generated during peeling of the inner shell membrane (ISM) of a 12-day-old chicken embryo and a 3-week-old raw egg. Various force profiles were measured from the two experiments and were collected as preliminary results [63].

It was verified that the novel 2-DOF force sensor using tri-axis optical fibres that each had a single-point FBG could be incorporated into and used with vitreoretinal surgical instruments, and the device demonstrated great potential for high sensitivity to lateral forces. Moreover, the device was able to measure the force magnitudes and could define the direction of the dominant force components. However, the device unable to measure the desired axial forces, which were considered noise and were eliminated from the design.

In the common epiretinal membranes peeling procedure, surgeons are required to operate the instrument deliberately while, keeping the forces at the minimum. At the same time, they visually monitor local surface deformation that may indicate undesirable forces between surgical tool and tissue. This hand-eye coordination complicates the procedure as it takes time to detect, assess and react to the faint cue.

The requirement for micron-scale manoeuvres while safely applying forces (below human perception) to delicate tissues was the main motivation for developing tactile tool-tip force sensor. The tool-tip force sensor should provide real-time force information which gently guides the operator towards lower forces during peeling task.

Physiological human hand tremor is one factor causing instrument velocity variations, where undesirable forces could occur. Several approaches have been introduced to reduce its effect and improve surgeon's fine motion control. One example is MICRON; a hand held device that uses piezo-actuators to counteract undesirable hand tremor and scale input motions [64]. Another example is to cooperatively controlled steady-hand using a device named “Eye Robot” to provide stability and precision [65]. Robot-assisted microsurgeries will reduce hand tremor and make the applied forces more controllable, and with integrating tool-tip force feedback limitations can be set such that the manipulator cannot apply forces beyond the limit.

Uneri *et al.* [66] designed, built and tested a platform for robot-assisted vitreoretinal surgery; they developed a novel cooperative control method that assists the operator in manipulating tissue within defined force limits. The previously designed 2-DOF force-sensor of the vitreoretinal surgical instrument (with simple hook) was mounted on the developed cooperative control robot with a remote centre-of-motion mechanism (RCM). The function of the developed cooperative control robot was to assist challenging retinal membrane peeling procedures, which required the surgeon to delicately delaminate fragile tissue that is susceptible to haemorrhage and tearing from undesirable forces. This combination allowed the researchers to directly measure the forces at the tooltip and use this information to limit the forces applied to the retina. The report described a series of experiments on the inner shell membrane of raw chicken eggs; the membrane was used as a simulation model with the aim of identifying and controlling the forces associated with peeling operations. The force sensing of the 2-DOF tooltip was adequate for the predefined manipulation tasks used in the controlled experimental setup.

Balicki *et al.* [67] combined the previous platform which was done by Uneri *et al.* in [66]; with a newly implemented auditory sensory substitution system to specifically assist the membrane peeling procedure. Their goal in combining the auditory sensory substitution system with cooperatively controlled manipulation was to decrease the forces applied during the membrane peeling and to simultaneously minimise the time required to complete the task. They have employed three cooperatively control methods which modulate the behaviour of the robot based on operator input and/or tool-tip forces. The methods include; proportional velocity control, linear force scaling control and proportional velocity control with limits. The auditory signal, which depended on the tooltip force sensor level, was used to define three different safety zones. Forces beyond 7 mN were defined to be a danger zones with the potential for retinal breaking and tearing, as previously determined by Gupta [68]. In this zone, the audio signal was represented by a high constant “Beeping”, continuously constant low frequency Beeping was defined in between 1 mN and 3.5 mN, lastly gradient higher frequency Beeping when forces measured are higher than 3.5 mN until 7 mN. Researchers have identified sticky tabs from 19 mm Clear Bandage as a suitable and repeatable phantom for delaminating. For comparison free hand trial also has been included, five trials for each method with and without audio feedback have been carried out. It has been found that the trials in the two out of three methods (linear force scaling control and proportional velocity control with limits) audio signal helped to stabilize the applied forces and reduced the task completion time as can be seen in [67]. However, in the free hand trial and Proportional velocity method; the audio feedback has reduced the large forces but significantly increased time completion task.

Membrane peeling is mostly performed using with microforceps; therefore, Kuru *et al.* [69] subsequently utilised three optical fibres that each had a single-point FBG force sensor to be integrated into a microforceps. The researchers intended to design a 2-DOF single-point FBG tactile sensor, which could be used in membrane peeling procedures. The designed microforceps were aimed not only at achieving technical functionality but also at meeting the requirements of the surgical environment. Consequently, a customised disposable microforceps tool was used. Integrating the FBG force sensors into the customised disposable microforceps was a challenging task because using new sensors for every operation would not be a cost-effective solution. In addition, the tooltip must be rotated during membrane peeling surgery to grasp the membrane at the right angle; hence, the optical fibre could be easily damaged or blocked by the surgeon. Solving these two problems required the building of two mechanically decoupled functional parts. The first part was a force sensor module that consisted of FBG sensors attached to a hollow Nitinol tube, and the second part was a forceps mechanism that consisted of an actuating tube and the grasper. This design enabled reuse of the force sensor module while the grasper and actuating tube could be replaced; furthermore, a forceps mechanism in which with the handle which could be rotated while the sensor module remained stationary was designed. After calibration, the designed microforceps was able to measure forces in any direction in the x-y plane and with any orientation of the graspers relative to the tool; the measurements had an accuracy of 0.3 mN and a precision of 0.25 mN. The device could be used in both freehand and robot-assisted surgery.

Four types of experiments were carried out to evaluate the performance of the tool with robot assistance and a cooperatively controlled platform. Furthermore, to evaluate the effect of the auditory force feedback system (AFFS), the tooltip force signal was translated into an audio signal that could be heard by the operator in the same manner as previous discussion [67]. The four experiments were conducted on an inner shell membrane (ISM) and a chick chorioallantoic membrane (CAM); these membranes were used as a biological phantom because they have previously been reported to be a suitable model for vitreoretinal microsurgical instrument studies [62]. The four different methods to assess the effect of such auditory force feedback and robotic assistance were including: free-hand peeling with AFFS, robot-assisted peeling with AFFS, robot-assisted peeling with force scaling, and lastly combining the robot-assisted peeling with both AFFS and force scaling.

According to [69], high-frequency oscillations in the delaminating forces had appeared during the free-hand procedure and were attributed to the hand tremor. These high frequency oscillations were reduced by using the steady-hand robot-assisted. In the case of force scaling, a smoother force variations was provided; whereas the auditory feedback was beneficial for keeping the forces within the safe operation zone. The results demonstrated that only the combination of robot assistance, force scaling and auditory force feedback was able to provide relatively stable and limited forces during membrane peeling. In conclusion, the system was able to help the surgeon to stabilise the peeling forces and limit them below the specified danger zone of 7 mN throughout the peeling manoeuvers. However, throughout the membrane peeling experiments with either the designed sensorised hook or the microforceps, the operator tried to keep the tool perpendicular to the peeling direction. Thus, only lateral forces could have a substantial effect on the tool. This restraint was actually imposed to reduce the effects of the axial force components but is certainly impractical during real medical surgeries.

#### 3.1.2. Otorhinolaryngology (ORL) Microsurgery Applications

The delicate nature of bony and cartilaginous structures within the sinus and ear cavities was behind the motivation of developing smart instruments during surgical interventions. The term ossicles indicates the three tiny bones in the middle ear “malleus, incus and stapes”. They are responsible for transmitting sound vibrations in order accordingly from the eardrum to the oval window on the cochlea, where cochlea will further converts this signal to nerve impulses.

**Figure 2.**
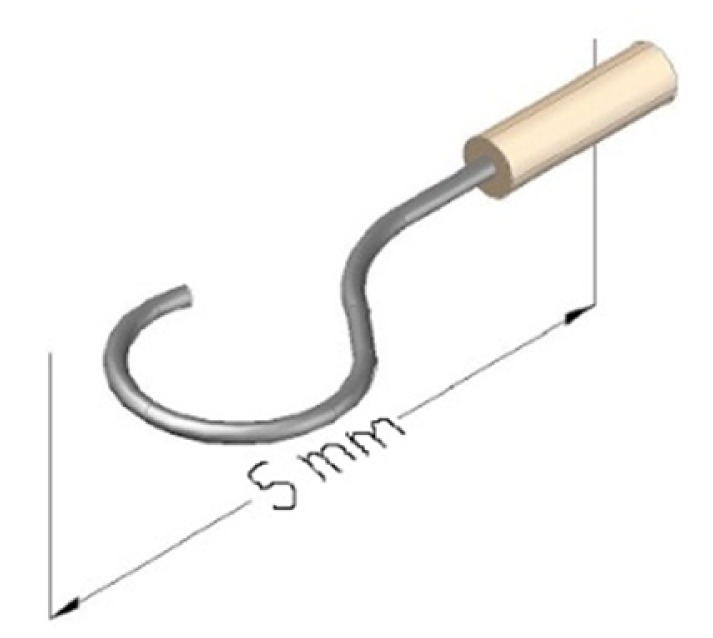
Schematic of the artificial stapes prosthesis which is usually clamped to the incus in the middle ear during surgery.

Abnormal growth of the ossicular bones that might occur around the stapes result in the loss of vibratory signal; as a result it causes mild to severe hearing loss (Otosclerosis). This is often treated surgically through intervention process called stapedectomy, the surgical removal of the stapes and replacement with an artificial prosthesis. Common procedure of this process involves microforceps to place one small crook shaped piston in the oval window connected to the incus through crimping thus bypassing the diseased stapes. The crimp process must be performed with the correct force measurement because loose crimps transmit sound poorly, whereas tight crimps will eventually result in necrosis of the incus. Therefore, developing an instrument that can provide the surgeon with force feedback during the crimping process is crucial. The main challenge was the integration of force sensing capability into the existence surgical tools. And to achieve most sensitive force measurement the location of the sensing element should be attached near the gripper where the cross section area is smallest. This requirement limits the selection of suitable sensors as the size in this region very small around 1 mm width. The conventional strain gauges cannot accommodate in such dimensions. Bell *et al.* [70] in their work have decided to choose FBG sensor for this application due to its potential small size, high sensitivity and other mentioned features. The reported work integrated a single-point FBG sensor into each of the two sides of a microforceps. The device was designed to measure the crimp forces during stapedectomy procedures. Figure 2 is showing the schematic of the artificial stapes prosthesis.

To minimise frictional effects on the measured crimp forces and to achieve high force sensitivity, the two FBG force sensors were placed near the gripper (indicating them as one in top and the other in bottom of the microforceps arms). Calibration was required to confirm the forces measured from the converted fibre deformation. The two wavelength shift signals detected by the spectrometer were converted to a deformation using known fibre parameters. Both are linearly related to the force and temperature as follows:

(6) $$S_{t}=FC_{t}+\alpha \Delta T$$

(7) $$S_{b}=-FC_{b}+\alpha \Delta T$$

Where *S_t_* and *S_b_* are the top and bottom FBG wavelength shift reading, respectively, while *C_t_* and *C_b_* are the inverse spring constant(calibration coefficients) of the top and bottom fibre sensors, respectively. The parameters Δ*T*, *F*, *α* are the temperature change, the applied force and the thermal expansion coefficient of the fibre, respectively. During calibration, the forces that were directly measured using piezoresistive sensor at the handhold and at the middle of the ring of the microforceps were compared with the forces measured by the FBG force sensor. The researchers determined the conversion factor using kinematic geometrical analysis to confirm the calibration procedure. They also related the forces measured by the piezoresistive element at the handhold with the calculated forces at the mouthpiece and those measured by the FBG. Both the top and bottom FBG were calibrated independently, and the calibration coefficients were determined by applying known forces. The researchers assumed that any temperature variation change would affect the two FBGs equally. Hence, the effect of temperature was eliminated. The repeatability was also checked by measuring the FBG signal-to-force relationship several times. The force applied was then calculated as a function of the wavelength shifts caused by both the upper/bottom fibre deformations and the determined calibration factors as follows:

(8) $$F=\frac{S_{t}-S_{b}}{C_{t}+C_{b}}$$

Several stapes prostheses were crimped in a clinically realistic scenario to test the designed force sensor for the microforceps. The initial testing was performed by crimping three prosthesis; two by a technician and one by surgeon on an excised incus fixed to the side of a dish in free space. Following these simplified crimps, an additional crimp was performed on a cadaver temporal bone (by the surgeon) [70].

The work was the first to demonstrate the attachment of a force sensor (with dimensions of only 1 mm × 2 mm) into the teeth of the microforceps for measuring forces in real time during a stapes crimping procedure. The two trials using the excised incus and the cadaver temporal bone are carried out and the results showed that the peak crimping force ranged from 2.4 N to 5.2 N during the two crimps trials. Additional artefacts during manipulation in trial of cadaver temporal bone were noted and were attributed to the hand tremor effect. To prevent the effect of physiological tremors, the microforceps force sensor was attached to the developed 1-DOF actuator which mounts on a robot end effectors. Robotic integration of the force sensitive microforceps will provide a means of insuring clinical outcome independent of surgeon skill by providing controlled application of crimp force and forceps positioning.

#### 3.1.3. Catheterisation Applications

MIS operations encompass various surgical tasks ranging from conventional endoscopic/laparoscopic methods to new sophisticated MIS techniques. In such sophisticated techniques, surgeons use equipment varying from robot-assisted surgical platforms for abdominal surgery to computer-controlled catheters for catheter-based cardiovascular surgery [6]. In cardiac disease, such as atrial fibrillation (AF), radiofrequency (RF) cardiac ablation is commonly used for treatment. Surgeons commonly pass a catheter venously into the heart, delivering it into the left atrium through a trans-septal puncture. Once the contact of catheter tip-tissue occurred, RF current heats myocardial tissues via Joule effects to temperatures in excess of 50 °C, producing transmural necrosis. In AF procedure often the goal is to isolate electrically the pulmonary veins where the sources of AF reside [71]. The success outcomes of this MIS are entirely dependent on full transmural lesion formation for electrical isolation [72], which in turn depends on steady contact level between catheter tip and target tissue. Contact level dependency interpreted as Inconsistent tissue contact may result in incomplete lesion formation that could result in AF recurrences, and too much contact level may result in tissue injury, which may lead to complications. In the absence of catheter tip-tissue contact force (CF) sensing surgeons are required to assess tissue contact using imaging-guidance or any indirect method, in which surgeons due to lack of soft tissue contrast in available image tracking might accidently push the catheter through the heart wall by applying excessive forces, resulting in a perforation problem. Current mapping techniques include; X-ray fluoroscopy and endocardial echocardiography recordings are both provide visualization, but they both provide little information about the contact level of the catheter-endocardial and time consuming. Furthermore, fluoroscopy increases the risk of malignancy to both patient and surgeon [73]. Therefore, several catheters ablation have been developed to be incorporated with CF sensors to monitor and measure direct CF in RF ablation including; reported study in [74] which modelled and analysed the resistance between the catheter tip electrode and the dispersive electrode during radio-frequency cardiac catheter ablation for the prediction of myocardium-electrode contact. Another research has incorporated two optical fibers into the catheter ablation to assess various parameters such as contact level, lesion quality, and char formation, *etc.* by comparing the transmitted and received NIR radiation spectrum [75]. The accuracy of these surrogate measures has not been extensively validated. Recently two types of ablation catheters were developed using three fibers with single-point FBG tactile sensing element to allow measuring and visualization of CF between catheter and cardiac tissues. The two types are TactiCath which was developed by Endosense SA (Geneva, Switzerland) in collaboration with Stanford University [76], and the THERMOCOOL SMARTTOUCH Catheter which is developed by Biosense Webster, Inc. (Diamond Bar, CA, USA) [77]. Several experimental studies and researches have been carried out by this device for measuring CF validation and assessing other parameters relating to CF. It has been shown that by being able to measure/monitor the CF; pressure can be controlled by surgeons and just apply the right amount to create transmural lesion while avoiding steam pops. Yokoyama *et al.* [38] has developed 7F open-irrigated catheter with 3.5 mm tip-electrode to measure contact force during RF ablation. They have incorporated 3 optical fibers with single-point FBGs of each to measure micro-deformations that correlate with force applied to the TactiCath catheter tip. The work purpose was to validate the accuracy of CF sensor (bench test), and to determine the relationship between the CF and tissue temperatures, lesion size, steam pop, and thrombus during RF ablation using a canine thigh muscle preparation. The catheter had a central lumen for saline irrigation during ablation and 6 small irrigation holes at the tip of 0.4 mm diameter around the circumference. The tip-electrode contains a thermocouple to measure the electrode temperature (ET). Two catheters were calibrated externally against a certified balance (force sensor type 5 N/0.001 N, Mecmesin, Slinfold, West Sussex, UK) with three force angles (axial to catheter 90°, lateral to catheter 45° and parallel to catheter 0°) to verify accuracy and sensitivity of CF FBG sensors. In repeating fashion of at least 10 times of each angle force, the CF readings were recorded from both calibrated balance and the catheter CF FBG sensors; while the catheter was being advanced to the balance gradually with force ranging from 0 to 50 g and *vice versa*. This up-down calibration sequence was to ensure equal distribution of the force samples.

The researchers have conducted experiments on ten anesthetized dogs, where their skin edges were raised over their thigh muscle to form cradle and superfused with heparinised blood (to prevent blood clots) at 37 °C. With this preparation the developed FBG ablation catheter sensors was held perpendicular to the muscle at CF 2, 10, 20, 30, and 40 g. RF energy was delivered for 60 s at 30 or 50 W with irrigation flow of 17 or 30 mL/min respectively at peak flow velocity 0.1 m/s, to simulate ablation sites with low local blood flow. Four 0.3 mm diameter fluoroptics temperature probes were included to have two positioned on opposite sides of the electrode-tissue interface to measure the temperature of the electrode-tissue interface; whereas the other two were bundled together with shrink tubing and inserted into the thigh muscle adjacent to the ablation electrode. The depths of the two probes below the thigh muscle surface were 3 and 7 mm to measure the tissue temperature every 125-ms intervals. For each of the five CF levels1 RF application of 30 W or 50 W was applied on all of the 10 dogs (on five sites along surface of the left side thigh muscle and right thigh muscle) and after every RF application, ablation electrode and the electrode-tissue interface were examined for thrombus. Then removing, cleaning and positioning the electrode catheter at a new site for another RF application. The root-mean-square power and impedance, and the ET, electrode-tissue interference temperatures were monitored continuously and recorded. A total of 100 RF applications were observed and statistical analysis was performed using SAS software. Two-Factor repeated analysis of variance (ANOVA) was used to determine/assess the significance of the relationship between the different levels of the CF and the initial impedance, impedance decrease, ET, electrode-tissue interface temperature, tissue temperature at depth of 3 and 7 mm, and lesion size.

Results from calibration verification have shown that the CF measured by FBG sensors at the tip of the catheter highly correlated to the ones measured by calibrated balance (R^2^ ≥ 0.988) for the two catheters (Table 3 show results for catheter #1). As for testing the impact of CF on RF lesion formation in the canine thigh muscle preparation, results confirmed that CF is a major determinant of RF lesion size, where larger and deeper lesions was produced by lower RF power (30 W) and greater CF (30 to 40 g) compared with lesions produced at high power (50 W) but lower CF (2 to 10 g). However, steam pop and thrombus incidence was also found to be increased with CF. Therefore, the ability to measure CF before the onset of an RF application would allow the selection of an appropriate RF power and application time to maintain efficacy (deep lesion) and minimize risk of steam pop and thrombus [38].

The limitation of this study is that it wasn't performed on a beating heart, where the catheter incidence angle or electrode-tissue contact area might have different results. In addition, the correlation of ET and electrode-tissue interface temperature would be very difficult in a beating heart because of limitation in measuring interface temperature and tissue temperature.

Despite the various capabilities of currently available technology, there has yet to be any data establishing how cardiac perforation can be reliably predicted. Methods that allow detection of perforation or even a warning of impending perforation will be a valuable asset for cardiac RF ablation.

**Figure 3.**
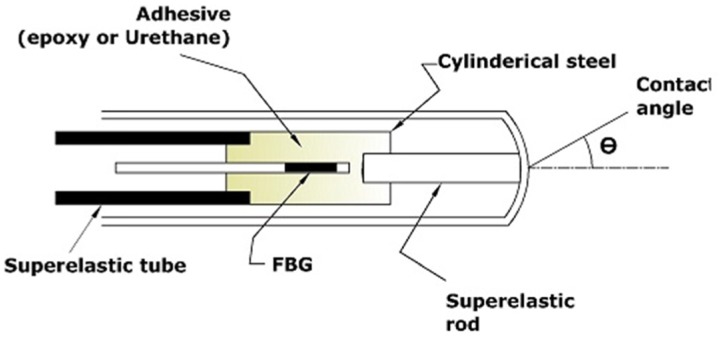
Schematic of the sensor head of the catheter.

A previous study [78] reported the feasibility of utilising a single-point FBG tactile force sensor to determine the catheter-endocardial contact level and predict the pre-perforation contact level during RF cardiac ablation. Two catheters, each with one FBG-based sensor, were developed separately in a form similar to that of ablation catheter. Both catheters had an FBG sensor that was bonded into a steel cylinder using an adhesive, and utilised a different type of adhesive (epoxy and urethane) to test their respective strain transfer behaviours. The designed catheter sensor and prototype schematically are shown in Figure 3. Two experiments were conducted on two male sheep to evaluate performance of their prototype sensors. The wavelength shift of the FBG sensor described in Equation (2) will be either a red shift (tensile strain) or a blue shift (compressive strain) corresponding to the dominant force components, which can be either lateral or axial. Thus, the researchers preferred to keep the catheter perpendicular to the heart wall surface as much as possible while the catheter was pushed through the heart wall to eliminate the lateral forces. For the initial part of the experiment, the catheter was alternated between resting in the space of the chamber and perpendicularly pressing against the endocardial surface.

Towards the end of the surgery, the catheters were pushed through the wall of the left atrium to induce mechanical perforation of the left atrial myocardium. The contact was initiated once the sensor was pressed firmly against the endocardial surface, and a periodic signal in the heart beating rhythm was observed. The epoxy-bonded FBG sensor exhibited less sensitivity than that of the urethane-bonded FBG; thus, all the disturbances due to vibrations from other body parts were filtered out, making the signal clearer when epoxy was used. Furthermore, the perforation was identifiable by a baseline-shifting loading phase followed by a rapid return to the prior wavelength levels. Thus, the use of an FBG sensor in catheter ablation enabled monitoring of the catheter-endocardial contact force and examination of any signal behaviour that facilitates predictions of transmural perforation of the left atrium. However, the superposition of the lateral force components with the axial ones induced noise during the experiments, mostly when the catheter buckled or for a high contact angle θ, as shown in Figure 3.

### 3.2. FBG Array Tactile Sensors (Distributed FBG Tactile Sensors)

In addition to measuring force amplitudes, tactile sensors are must be able to detect and measure the distributed force profile of the contacted object under a dynamic and static load. This capacity enables MIS/MIRS surgeons to assess and characterise the contacted tissues. The information that can be retrieved from the distributed force profile includes the following: concentrated force position, relative hardness of the contacted soft tissue, and shape of the tool deflection.

The multiplexing ability of FBG sensors was behind the motivation of utilizing it as it facilitates the straightforward measurement of the distributed forces; because each FBG has its own independent wavelength. Moreover, the multiplexing facilitates the attachment with other objects and makes the wiring simpler than that of other types of electrical/optical small tactile force sensors because one single fibre can have many FBG sensor elements as long as there is sufficient interrogator bandwidth.

**Figure 4.**
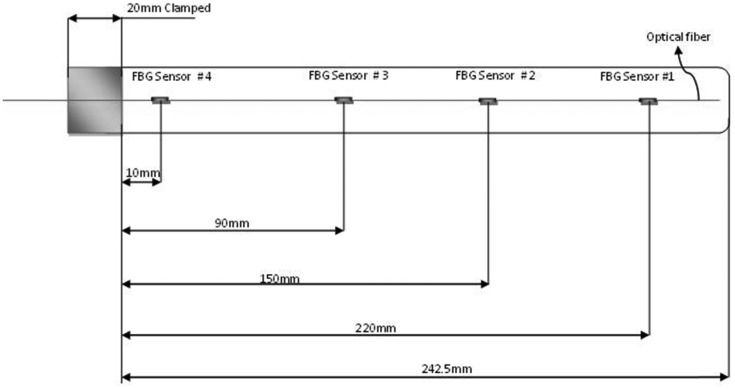
Layout of 4 FBG array sensors on a 1-D tactile sensing strip [79].

**Figure 5.**
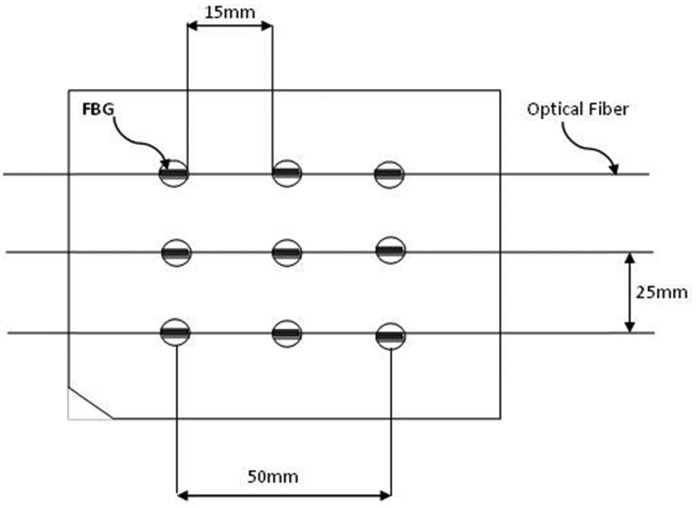
Layout of 2-D 9 FBG sensor array in a 3 × 3 grid for distributed tactile sensing on a surface.

Distributed FBG tactile sensors can be designed in a one-dimensional (1-D) or 2-D pattern, as shown in Figures 4 and 5, respectively.

#### 3.2.1. Endoscopy and Laparoscopy Applications

Advances in robotic systems have led to their use in teleoperation rooms which meant there will be a physical separation between surgeon's hands and surgical instruments, resulting in a loss of force feedback. Therefore, researchers are presently attempting to add a tactile sense to these robots for use in teleoperation manipulators and haptic interfaces.

In applications such as laparoscopic surgery, grasping instruments are used to manipulate tissue. The surgeon should be able to measure the tissue properties and feel their variations through the instrument; as tumour or any other hard structures which buried under the tissue surface may not be seen intraoperativly by the endoscopic camera or any other visualize devices [80]. Furthermore, secure grasping such as for biopsy in endoscopic and laparoscopic is a crucial. Conventional force sensing devices, such as strain gauges load cells are commercially available for robotic and industrial applications. However, limitations such as; sterilizability, disposability, their big size and incompatibility with other medical appliances (*i.e.*, MRI) have made the electrical-based sensors difficult to be adopted for MIS. Researchers at Aston University (Birmingham, UK) led by Cowie *et al* in [79] have developed a first generation sensor design using optical fibre sensor FBGs. The project was as a part of a research project to ascertain whether FBGs could be used to provide an endoscope with tactile sensitivity [79]. In that investigation, the researchers used a steel strip attached to it a four FBG sensor array with only a single fibre connection. Another identical steel strip was utilised with four resistive strain gauges fitted to this strip in the same positions with eight connections. These strips were used to assess the load/force position and magnitude; the two results were then compared. The performance of the FBG sensors was found to be more accurate than that of the strain gauges. The researchers also investigated a 2-D stainless steel plate with nine FBG sensors in a 3 × 3 grid arrangement to establish a “smart surface”. An identical steel plate with 16 infrared displacement sensors was used for comparison. Neural networks were trained to process the sensor data from both systems to detect simultaneously the position and shape of the contacted load in real time. Four shapes of equal area and weight were placed iteratively on top of the surface at different positions. The results showed that the smart surface using the distributed FBG tactile sensors outperformed the infrared sensors with less rms error in detecting the load position. Furthermore, the system using the distributed FBG sensors was able to detect the shape at any position with an accuracy of 91%. The investigation demonstrated that distributed FBG tactile sensors offer advantages over other sensor types, including the ability to use fewer connections, the immunity to electromagnetic noise, the ability to be embedded within materials without loss of material strength and the ability to operate at high temperatures. However, the established grid cannot be used in MIS due to its large dimensions (low spatial resolution). This grid has been mentioned here to show the possibilities for a smaller grid with high spatial resolution. This type of grid is possible in practice with shorter FBGs and can therefore be attached to graspers for tissues characterisations and for measuring gripping forces.

**Figure 6.**
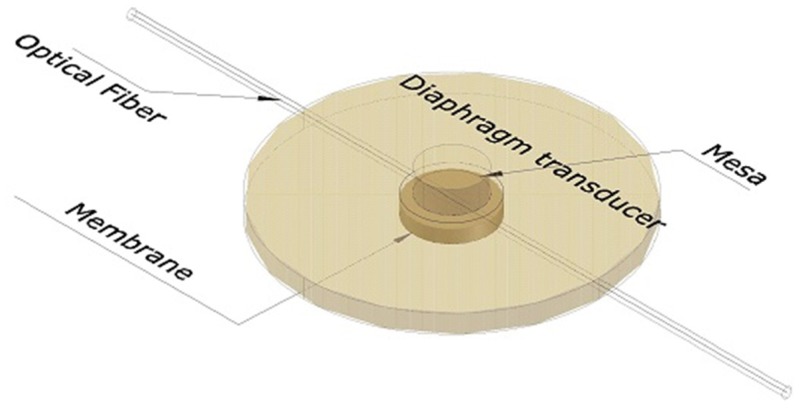
Taxel prototype of the designed diaphragm-type transducer.

**Figure 7.**
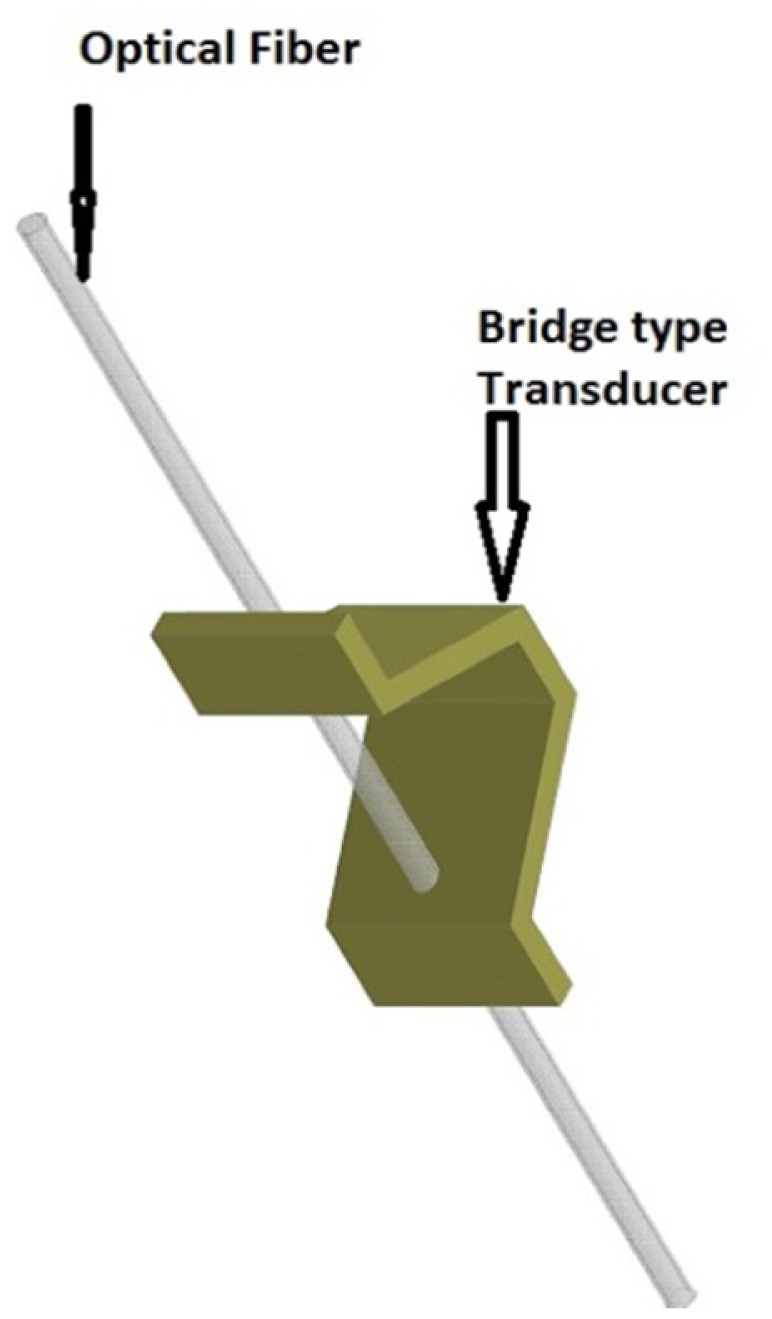
Taxel prototype of a bridge-type transducer.

Heo *et al.* [33] designed, fabricated, and evaluated two types of FBG array tactile sensors with a 3 × 3 grid of taxels to detect distributed normal forces. The first type used a diaphragm-type transducer, while the other used a bridge-type transducer. The diaphragm transducer taxels were made of PDMS (poly-dimethylsiloxane) and their contact mesa and membrane design schematically is shown in Figure 6. The FBG sensor was embedded in the membrane. Thus, when an external force was concentrated on the contact mesa, the membrane was deflected, inducing elongation in the FBG sensor; a wavelength shift was thus detected. In contrast, the bridge transducer taxels were made of beryllium copper (BeCu), which resembles a bridge, as shown in Figure 7. When external normal forces were applied to the top of the transducer, the bridge extended symmetrically in the direction of the optical fibre, which was attached to the rim of the transducer. Thus, the FBG sensor elongated, and a wavelength shift was detected. The distributed FBG tactile sensor of the diaphragm technique was developed to create a large area tactile sensor that has good sensitivity but low spatial resolution, similar to human body skin. The FBG sensor that was used in each taxel had a length of 10 mm, and the taxels were arranged in a pattern with 25 mm spatial resolution (see Figure 5). In contrast, the bridge-type distributed FBG tactile sensor was developed to create a small area tactile sensor that has good sensitivity and spatial resolution, similar to finger skin. The FBG sensor in this type of sensor had a length of 2 mm. Its taxels were arranged in a grid with 5 mm spatial resolution, including the length of the transducer taxel and the FBG length. Three optical fibres were used in each type of tactile sensor with three FBG sensors located in each fibre; every FBG sensor element has its own wavelength. During the design, the researchers accounted for the effect of the non-uniform strain distribution along the FBG length that might occur for the diaphragm type sensor. A non-uniform strain distribution would lead to a distorted Bragg wavelength (chirping) signal, which would decrease the measurement accuracy [81]. In addition, bending of the optical fibre was avoided to maintain the signal strength. However, the bridge tactile sensor has a structure designed to extend only in the fibre direction and will therefore not have micro-bending. Thus, the Bragg wavelength shift occurs without chirping or light loss. Both prototypes compensated for temperature effects by including an FBG sensor reference to detect temperature changes.

During the individual taxel evaluations, the prototype sensors were calibrated using a verified uniaxial load cell. The repeatability was evaluated as well using three iterations of same loading conditions for the specified prototype force sensor. The hysteresis was also tested. The accuracies for both prototype taxels were found to be 99.9%, and the resolutions were approximately 0.005 N in the diaphragm-type sensor and 0.001 N in the bridge-type sensor. The relationship between the applied forces and wavelength shift in diaphragm-type sensor was revealed to be nonlinear due to the non-linear material property of the transducer; the nonlinear curve fitting was as follow:

(9) $$Y=1.225\times 10^{-4}+0.07939X+0.0126X^{2}$$

Where *Y* & *X* are the wavelength shift and the applied force respectively.

The performances of the two types of 3 × 3 distributed tactile sensors were experimentally verified by applying a distributed force and a point force. The shifts of each Bragg wavelength were measured using a tuneable Fabry–Perot filter, which controlled using a LabView program. Therefore, the shifts of each Bragg wavelength, which represented the change in distributed force, could be detected simultaneously through this interrogation system using wavelength division multiplexing (WDM). The distributed force was measured directly by using the evaluated calibration coefficients.

The experimental tests showed that both types of FBG array tactile sensors had good performance, good sensitivity, repeatability and no hysteresis. However, the proposed sensors were somewhat larger than the MEMS-based small tactile sensor arrays, and the system was expensive due to the interrogator system. FBGs of shorter length could increase the spatial resolution of the sensors and make smaller sensors possible.

**Figure 8.**
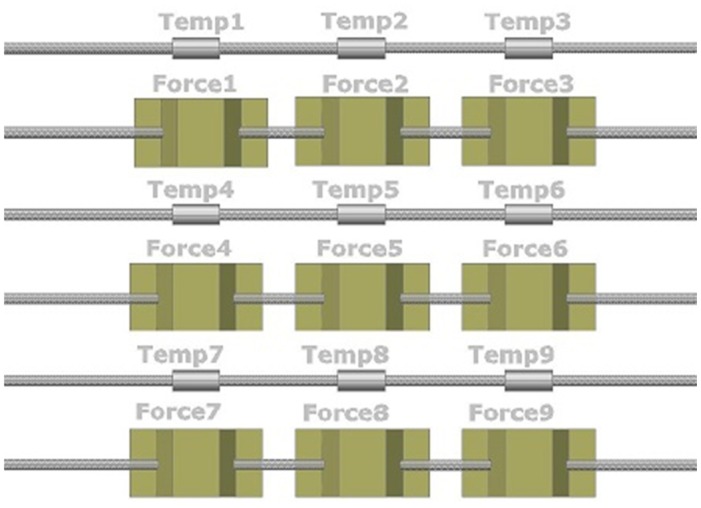
The fabricated two-dimensional sensor array that can detect distributed forces and distributed temperatures.

Later in the same year, Heo and Lee [82] designed and fabricated a distributed FBG tactile sensor that was able to simultaneously detect a distributed temperature and force. The researchers utilised the previous bridge-type 3 × 3 force sensors [33] in the newly designed 3 × 3 temperature sensors. The FBG temperature taxel was covered by a flexible tube to prevent the Bragg wavelength from being affected by any thermal strain from the attached material. The taxel used an FBG that was 2 mm in length; this size was identical to that of the tactile force sensor. The FBG was calibrated by inserting one FBG taxel along with a thermocouple into water that was heated by a hot plate. The output of the thermocouple signal and the wavelength shift caused by the temperature change were compared, and linear relationship was found. The prototype taxel was found to be 99.8% accurate, and the resolution of this fabricated temperature taxel was approximately 0.1 °C. Curve fitting indicated that the relationship between the wavelength shift and the temperature was an approximately linear polynomial equation, and the calibration coefficients were determined. Then, a distributed tactile sensor consisting of nine FBG temperature taxels was created and calibrated using tactile force sensors, as shown in Figure 8.

**Figure 9.**
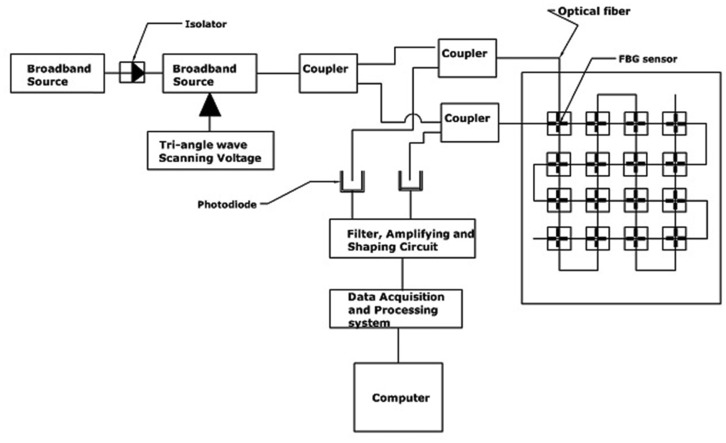
A schematic diagram of the signal detection using three optical splitters for a parallel detection scheme that simultaneously measured the distributed temperatures and forces.

For this arrangement of taxels with each taxel having its own wavelength, the researchers managed to accurately calculate the external normal forces by excluding the temperature effects, which could be measured using the temperature sensors. The verification utilised a scale calibration weight of 200 g; the surface temperature of the weight was higher than the room temperature (nearly 60 °C compared to approximately 20 °C), and the weight was loaded on force taxels Nos. 1, 2, 4, and 5, as shown in Figure 9.

The experimental results indicated that an accurate distributed force change that compensated for the temperature effect could be obtained as along with a distributed temperature change using the temperature sensor array. However, the overall area of the sensor was enlarged, and number of fibres also increased, as shown in the arrangement of Figure 8.

Recently Junjie *et al.* [83] designed and evaluated a composite tactile force and temperature sensor in a 4 × 4 grid of FBG array sensors. The sensor should be able to measure simultaneously the force and the temperature of the contact object. The researchers adopted an elastic bridge-type beam for each sensor element/taxel, and two crossed smooth grooves were made on the bridge beam. One was made transversely, while the other was longitudinal with depths of 0.15 mm and 0.3 mm, respectively. Two identical FBGs were bonded into these two grooves, with one to sense both the force and temperature of the contacted object (transverse groove) while the second was only affected by the temperature (longitudinal).

The device consisted of sixteen sensor elements in 4 × 4 arrays of tactile sensors. Two optical fibres were used, and each had an array of sixteen FBGs. All the FBGs that were bonded on the transverse grooves were cascaded into one single-mode fibre; each had its own wavelength, which should not overlap with that of the others. However, the second optical fibre contained the other cascaded FBGs, which were bonded on the longitudinal grooves in an identical manner. The researchers utilised a parallel detection scheme to interrogate and detect the peak points of the different wavelengths of the two channels. A broadband light source with a span of 50 nm and centre at 1,550 nm was used along with a tuneable Fabry-Perot filter, three 2 × 1 couplers, and two photodiodes, as shown in Figure 9. The wavelength shift signals of each channel were detected by photo-diodes and converted into voltage signals; the signals were then filtered, amplified, and finally shaped into a rectangular impulse, which was acquired and processed using a field programmable gate array (FPGA) processor. The Bragg wavelength shifts of the FBGs were observed and plotted via a computer demodulation program to simultaneously calculate the contact force and the contact temperature of the sensor array.

The FBGs that are affected by both strain and temperature can be corrected for temperature effects, which will be measured by the longitudinal FBGs. The researchers demonstrated that a contact force resolution of 0.01 N and a contact-temperature resolution of 0.5 °C could be achieved using the parallel method of interrogation. Compared with Heo's work [82], this work achieved better spatial resolution with fewer fibres connections. The results showed sufficient performance and the possibility to practically utilise a composite tactile sensor array. However, the article mentioned neither the dimensions of the taxels nor the length of FBG sensors. The researchers only stated that there is a possibility for improving the spatial resolution.

Operating complicated tools and performing delicate tasks using robotic surgery require a manipulator of great precision and coordination. Grasping tissue is crucial during laparoscopy or biopsy procedures. An overly strong grasp can cause traumatic injury to the tissue, while the tissue can be dropped from a too loose grasp. Therefore, force sensing is one of the most critical requirements for this type of robot control. An embedded array of FBG tactile force sensors can be used to measure and localise the applied forces.

In a research led by Park *et al.* [84], the research group designed, fabricated and investigated FBG arrays force sensors that were embedded in a polymer-based structure. The work aimed to create a lightweight, rugged appendix for robots that featured embedded sensors to enable the robot to measure grasping forces in real time and to estimate the contact location during dexterous robotic manipulation tasks. The structure was designed to resemble a human finger with a hexagonal exoskeletal shape to reduce the weight while maintaining high strength. Four FBG force sensors was determined to be the optimal number for the structure, and finite element analysis (FEA) indicated the positions where the sensors could be placed for the most concentrated strain distribution. Static force experiments on the sensorised structure revealed minimum detectable force changes of less than 0.02 N and practical force measurement resolutions of less than 0.15 N. In contrast, for the dynamic test revealed a dominant frequency of the finger structure at 167 Hz.

The sensorised structure could provide better sensitivity and resolution if a stiffer structure was used. Furthermore, a greater number of sensors will allow for more accurate force localisation. However, the finger sensors are capable of resolving small forces and are immune to electromagnetic disturbances; therefore, the system can be mounted on a robot-assisted surgical platform for an MRI environment. Later in 2009, the same group developed a single-axis closed-loop hybrid force/position control system to utilise the sensorised exoskeletal finger prototype [85]. Thus, the robot-assisted system was able to control the exerted forces during manipulation such that the forces did not exceed the predefined force limit. The rms of the force errors during the force control was <0.03 N.

#### 3.2.2. FBGs in Minimally Invasive Treatments and Biopsy Applications: Shaping Sensing

**Figure 10.**
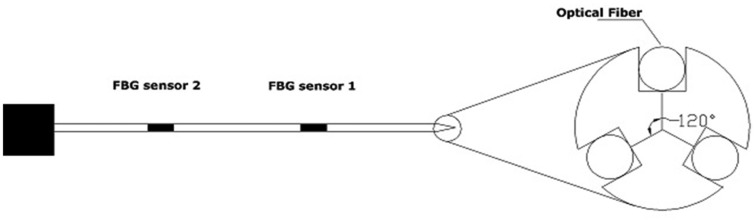
Schematic diagram illustrating the modified needle prototype with the 3 incorporated optical fibers each associated with two FBGs.

During procedures such as diagnostic biopsies or localised treatment in impalpable areas (e.g., the prostate, breast or lung), physicians combine imaging procedures (e.g., MRI) with a needle biopsy for guidance. The function of imaging is to make sure that the needle does not deviate from the planned trajectory and that the needle reaches the correct spot. However procedural complications, low spatial resolution and relatively low contrast resolution make it difficult to precisely identify the needle tip deflections. To tackle the drawbacks of the imaging procedures, distributed tactile force sensors can assist physicians indirectly by allowing them to view the needle head route in tissues in real time. Needle shape can be reconstructed based on strain measurements within the needle. FBG array sensor technique has shown very strong candidate for this particular application, where it enables for needle shape reconstruction in real-time with trivial error in tip-deflection accuracy. Experimental study with needle integrated by three optical fibers with two FBGs each have demonstrated that; during needle tip deflections up to 12.5 mm, the tip position was estimated with a mean accuracy of 0.89 mm (standard deviation of 0.42 mm) [86], where this accuracy is appropriate for applications such as RFA of liver tumors. Another investigation reported in [87] has presented a prototype of a flexible Nitinol needle (Ø 1.0 mm and length 172 mm) integrated with an array of 12 Fiber Bragg Grating (FBG) sensors. They performed experiments where the needle is inserted into a soft-tissue simulant, and the 3-D needle shape is reconstructed using the FBG sensors. They compared the reconstructed needle shape to deflection obtained from camera images and their models. The maximum error between the experiments and the camera images they found was 0.74 mm. Here we will discuss in more details a work that has been demonstrated even better accuracy with less tip deflection error. Park *et al.* [88] modified an 18-gauge MRI-compatible biopsy needle for incorporation into distributed FBG force sensors. The 18-gauge needle is a typically sized needle used for MRI-guided interventions, such as those for prostate and breast biopsies. The needle is composed of two parts, inner and outer, and has a length of 150 mm. The inner part (stylet) is solid with a diameter of 1 mm, while the outer part is a hollow thin sheath. Both are made of a non-magnetic nickel-cobalt-chromium-molybdenum alloy. Three square grooves of 350 μm were made using electrical discharge machining along the stylet axis at 120° intervals. Three optical fibres that were 250 μm in diameter were utilised; each fibre had an array of two FBGs force sensors, and the fibres were attached to these grooves to measure the distributed forces along the needle as shown in Figure 10. The work aimed to track the needle bending deflections at greater bandwidth and accuracy than those available when only viewing the tool with MRI.

A broadband light source was used along with wavelength division multiplexing to simultaneously interrogate and compute all the wavelength shifts of the returned light. Then, the prototype was connected to a computer for calibration and visualisation. Using some assumptions, the authors analytically modelled the needle as a slender cantilever beam that was supported at one end and subjected to radial and axial forces. The temperature was assumed to be uniform across the needle because of its extremely small diameter; thus, an FBG for temperature compensation was not added. To determine the minimum number of FBG sensors required to sufficiently capture the needle profile even for complex force distributions, the researchers developed a simulation of a Fourier series model with eight, four and two terms. The simulation was conducted to approximate the possible combinations of applied forces, including a distributed force profile along the needle and a somewhat concentrated force near the tip. To assess the needle tip location errors that may be expected in the computed profile, the researchers utilised a Monte Carlo simulation of various possible applied force distributions at any needle orientation in the (*x*, *z*) plane; thus, they were able to determine the best location for the two sets sensors that would produce the smallest tip location errors. The needle prototype was calibrated for 3-D bending using two digital cameras with a resolution of 0.05 mm/pixel and a maximum optical distortion less than 0.35%. The two cameras were fixed in two orthogonal planes, and various needle deflections were applied in the (*x*, *y*) and (*z*, *y*) planes while images were recorded using two cameras. Then, the images were processed to obtain the profile of the centreline of the needle. Three experiments were conducted for calibration procedures to determine the calibration matrix at each sensor location; the experiments included changing the temperature at the sensor location with no mechanical loading to determine temperature effects.

The following maximum errors were found in the local curvature measurement from the six sensors: 2.14%, 0.14%, 0.65%, 0.27%, 0.35%, and 0.70% in order of increasing sensor number for the *x*-axis loading and 0.06%, 0.19%, 0.21%, 0.05%, 0.19%, and 0.18% in order of increasing sensor number for the *z*-axis loading. Sensor number one showed a relatively large error during the x-axis loading, and the error was attributed to a manufacturing error in the FBG placement in the needle. Using the calibration matrix for each sensor location allows for calculation of the local curvature from the real-time sensor signal during the procedures. The researchers found that this approach yielded tip deflection errors with rms values of 0.38 mm(*x* − *y* plane) and 0.28 mm (*y* − *z* plane) when the actual deflections were in the range of ±15 mm. The errors slightly decreased as the actual deflection range decreased. The integrated system included a diffraction-grating-based FBG interrogator with an update rate of 4 Hz, LabView and Matlab scripts, and a calibration matrix. The researchers were able to monitor the real-time needle deflection and bend shape on a computer screen. The work demonstrated that the developed needle produced no adverse imaging artefacts when used with an MRI scanner and that the strong magnetic field did not degrade the signal. The estimated needle deflection acquired by the distributed FBG tactile sensors was compared to the deflection measured in the MR images. The needle was placed in a water bath and deflected with nylon screws in five different loading configurations. The results showed that the estimated tip deflections were comparable to the deflections measured in the MR images. A summary discussion is carried out in Table 4 which compares between the designed FBG sensors according to their specific MIS applications.

## 4. Conclusions

Detecting the interaction forces between surgical tools and tissues in MIS or microsurgery applications is important for better performance and timing. The significance of these forces can be addressed with the use of newly developed fibre Bragg grating miniaturised sensors, which have been discussed.

The paper presents a summary of the previous and the current state-of-the art FBG tactile force sensors. It is obvious that attention has been given specifically to FBG for microsurgery and MIS due to its numerous benefits, especially its compatibility with MRI and its small size. Two types of tactile techniques were discussed a single-point FBG tactile sensor a distributed FBG or tactile array sensor.

The two configurations of the arrays of FBG tactile sensors that have been discussed in the present paper include a 1-D array and grids of 2-D arrays. The distributed FBG tactile sensor with a 2-D grid was found to be promising; however, it is inappropriate for MIS applications due to the large size. To our knowledge, no articles on the minimisation of this type of structure have been published.

The array of FBG tactile sensors was also applied to an MRI-compatible biopsy needle [88]. Although the procedure was complicated, the three arrays of FBG sensors were able to convert the tactile information to the shape of the needle. Even with a small number of FBGs, the results indicated that FBG use will be promising in the biopsy applications.

Unlike other fibre optic sensors, the FBG tactile sensor has been shown to have a very broad operational sensing range that is well-matched to all the discussed different tissues types. For example, 1-DOF and 2-DOF devices that are used in simulated vitreoretinal surgery showed a good working force-range agreement with the determined forces in retinal surgery, as demonstrated by Gupta [68]. Furthermore, the discussed FBG tactile sensors used for measuring the peak interaction forces demonstrated adequate resolution and high sensitivity. Despite the complicated and challenging nature of the miniaturising process, which is required for the working environment, the literature has reported significant progress in the 2-DOF design [61,63,66,67,69]. All devices featured a single-point FBG tactile sensor that would enable the surgeon to monitor, control and stabilise the applied forces. Nevertheless, accommodating an additional sensor into the sensor device can post a challenge; the extra sensor must tackle issues such as temperature effects, as studied in a sensorized catheter [78]. However, our future perspective is to adopt the design of the 2-DOF, which was demonstrated by Iordachita *et al.* [63]. This design will be combined with a catheter device, which was introduced by Ho *et al.* [78], with some additional modification to the deformation part. Furthermore, the superelastic rod must be minimised to fit in the middle portion of the microsurgical tool.

The studied single-point FBG tactile sensors mostly focused on measuring transverse interaction forces, while the desired axial forces still pose a large and unresolved challenge. Normally, the stiffness of the tool shaft is greater along the axial direction than along the transverse direction; thus, a high-sensitivity sensor element is required. Designing 3-DOF, which is our goal in future, will enable separate measurements of the axial and transversal forces, leading to more accurate measurements; thus, the tools will become more practical and can consequently become commercialised.

## Figures and Tables

**Table 1.** Various FBG tactile sensors developed in MIS applications.

**MIS/MIRS Application**	**Design/Development/Technique**	**Group**	**Year**
Endoscope with tactile sensitivity	Array of 4 FBGs attached to steel strip	Aston University, Birmingham UK	2006
Radiofrequency (RF) catheter ablation for atrial fibrillation (AF)	3 Optical fibers with FBG each integrated into a catheter tip	University of Oklahoma Health Sciences Center & Endosense SA, Geneva, Switzerland	2008
Vitreoretinal microsurgery	1 FBG integrated into 1-DOF	Johns Hopkins University	Early 2009
Vitreoretinal microsurgery	3 FBGs into 2-DOF	Johns Hopkins University	Late 2009
Vitreoretinal microsurgery minimally invasive robotic surgery (MIRS)	Platform with cooperative control system for manipulating 2-DOF FBG sensor	Johns Hopkins University	2010
Vitreoretinal microsurgery & MIRS	Testing previous platform control with added auditory system	Johns Hopkins University	2010
Otorhinolaryngology (ORL)	2 FBGs integrated in top and bottom Microforceps' arms	ARTORG Center University of Bern	2010
Needle Biopsy and treatment	3 optical fibers with 2 FBGs each to monitor real-time 3-D needle deflection	Wyss Institute for Biologically Inspired Engineering, Harvard University	2010
Vitreoretinal microsurgery	FBG integrated into disposable Microforceps 2-DOF	Technische Universität München & Johns Hopkins University	2012
RF ablation and predicting perforation	1 FBG in designed catheter (2 catheters with two different adhesive bonding FBG inside the hollow.	University of Houston, Houston, TX 77004, USA	2012
Needle Biopsy Shaping sensing	3 optical fibers with 2 FBGs each integrated with needle biopsy	Delft University of Technology, Netherland	2012
Needle Biopsy Shaping sensing	Array of 12 FBGs integrated into Nitional needle biopsy for reconstruction the 3-D shape.	Delft University of Technology, Netherland	2013

**Table 2.** Wavelength/Force ratio *vs.* position along the tool.

**Position Relative to the Tool Tip (mm)**	**Sensor Sensitivity (Picometer/mN)**
1	13
5	9
10	4.2
12.5	3
14	1
15	0

**Table 3.** Correlation between catheter tip CF calculated from FBG sensors as a function of measured force by certified balance.

**Catheter Position Relative to the CF**	**R-Squared Value**	**Linear Fit**	**Mean Error (g)**	**Standard Deviation (g)**	**Sensitivity Error (%)**
Perpendicular (90 degrees)	*R*^2^ = 0.996	*Y* = 0.994*X* + 0.83	0.7	1.0	−0.6
Parallel (0 degree)	*R*^2^ = 0.998	*Y* = 0.989*X* + 1.24	1.0	0.7	−1.1
(45 degrees)	*R*^2^ = 0.993	*Y* = 1.028*X* + 0.22	0.8	1.4	2.8

**Table 4.** Summary for FBGs tactile force sensor in MIS.

**Author**	**MIS/MIRS Application**	**Design/Development/Technique**	**Sensitivity/Resolution/ Accuracy/Working Range**	**Meet Specifications?**	**Alteration, if any**
Cowie *et al.* [79]	endoscope with tactile sensitivity	Array of 4 FBGs and 9 FBGs in 1-D and 2-D resp. to detect load position, shape of contacted surface	Error ≈ 11mm (2.7% of full scale)	No. Big size cannot fit in MIS	Shorter FBGs and more sensors would improve
Yokoyama *et al.* [38]	RF ablation AF(Cardiac)	3 FBG into a catheter tip. (TactiCath Endosense SA))	Res. = 0.01N; W.R = 0–0.5 N	Yes	-
Sun *et al.* [61]	Vitreoretinal microsurgery	1 FBG integrated into 1-DOF (device)	Res = 14 pm/mN, sensitivity = 0.25 mN	Yes, but not applicable due to 1-axis forces only	Only if 3-axis and temp. compensated
Iordachita *et al.* [63]	Vitreoretinal microsurgery	3 FBGs into 2-DOF (device)	Sensitivity = 0.25 mN	Yes, but it lacks of axial forces and temp. compensation	Fourth FBG in the middle to decouple the axial and lateral forces
Bell *et al.* [70]	ORL	2 FBGs integrated in top and bottom Microforceps' arms	Peak measured = 2.4 N−5.2 N & sensitivity = 0.12 nm/N and 0.14 nm/N	Yes	-
Park *et al.* [88]	Needle Biopsy and treatment	3 optical fibers with 2 FBGs each to monitor real-time 3-D needle deflection	Max tip deflection error 0.38 mm in +15 mm actual range	Yes	Proper sensor locations with more FBG sensors could improve the accuracy
Kuru *et al.* [69]	Vitreoretinal microsurgery	FBG integrated into disposable Microforceps 2-DOF	Accuracy = 0.3 mN & sensitivity = 0.25 mN	Yes, but it couldn't measure axial forces	3-DOF required/by attaching FBG behind grasper stick to some slides attached with proper spring to induce compression/tensile onto FBG
Biosense Webster Inc. [75]	Catheter for RF ablation AF	THERMOCOOL SMARTTOUCH	NA	Yes	-
Ho *et al.* [78]	RF ablation andpredicting perforation	1 FBG in designed catheter (2 catheters with two different adhesive bonding FBG inside the hollow.	Threshold res. = 0.01 nm and 0.05 nm for epoxy and urethane adhesive resp.	No calibration was discussed to verify parameters such as force sensitivity	-
Henken *et al.* [86]	Needle Biopsy Shaping sensing	3 optical fibers with 2 FBGs each integrated with needle biopsy	Tip position was estimated with accuracy of 0.98 mm within range of ±12.5 mm	Yes	More FBG members will enhance accuracy
Roesthuis *et al.* [87]	Needle Biopsy Shaping sensing	Array of 12 FBGs integrated into Nitional needle biopsy for reconstruction the 3-D shape.	The maximum accuracy error of tip position with camera image was 0.74 mm	Yes/	-
